# Impact of Traditional Chinese Medicine therapy focused on strengthening the body on postoperative recurrence and metastasis prevention in stage IIIA non-small cell lung cancer: a real-world retrospective cohort study

**DOI:** 10.1186/s13020-025-01195-x

**Published:** 2025-08-29

**Authors:** Ruoyan Qin, Yi Jiang, Liping Shen, Jie Qian, Yanlan Kang, Rui Fan, Lingshuang Liu

**Affiliations:** 1 Department of Oncology, Longhua Hospital, Shanghai University of Traditional Chinese Medicine, Shanghai, 200032 China; 2 Department of Emergency and Critical Care Medicine, Shanghai Chest Hospital, Shanghai Jiao Tong University School of Medicine, Shanghai, 200030 China; 3 Academy for Engineering and Technology, Fudan University, Shanghai, 200433 China; 4 Department of Integrated Traditional Chinese and Western Medicine, Shanghai Pulmonary Hospital, Tongji University, Shanghai, 200433 China

**Keywords:** Non-small cell lung cancer, Disease-free survival, Neoplasm metastasis, Traditional Chinese Medicine, Cohort study, Chemotherapy

## Abstract

**Background:**

This study aimed to evaluate the impact of Traditional Chinese Medicine (TCM) therapy focused on strengthening the body on postoperative recurrence and metastasis prevention in patients with stage IIIA non-small cell lung cancer (NSCLC).

**Methods:**

This retrospective cohort study analyzed real-world data from patients with stage IIIA NSCLC who underwent radical surgery between January 2016 and January 2022 at Longhua Hospital, Shanghai University of Traditional Chinese Medicine. Patients were classified into an exposed group, receiving adjuvant chemotherapy or radiotherapy combined with TCM therapy, and a non-exposed group, receiving only adjuvant chemotherapy or radiotherapy. The primary outcome was disease-free survival (DFS), while secondary endpoints included 1-year, 2-year, and 3-year DFS rates (DFSR).

**Results:**

A total of 700 patients were included, with 340 in the exposed group and 360 in the non-exposed group. After propensity score matching, the exposed group demonstrated a significantly longer median DFS compared to the non-exposed group (32.0 months [95% CI 24.0–38.0] vs. 17.0 months [95% CI 15.0–20.0], *p* < 0.001) and higher 1-year, 2-year, and 3-year DFS rates (78% vs. 63%, 56% vs. 38%, and 44% vs. 24%, respectively; *p* < 0.001). TCM therapy was associated with reduced recurrence and metastasis (HR = 0.58, 95% CI 0.48–0.70, *p* < 0.001). Subgroup analysis showed greater DFS benefits following TCM therapy in patients with N2 involvement and those over 65 years (both *p* < 0.05).

**Conclusions:**

TCM therapy focused on strengthening the body may prolong DFS and improve DFSR in postoperative stage IIIA NSCLC patients. However, further large-scale prospective studies are needed to validate these findings.

## Background

Lung cancer is the leading cause of cancer-related morbidity and mortality in China, with an estimated 1.06 million new cases and 733,000 deaths in 2022 [1]. Non-small cell lung cancer (NSCLC) accounts for 85%–90% of cases, and surgery is the preferred treatment for early to middle-stage NSCLC (stages I–IIIA). However, despite radical resection, patients with stage IIIA NSCLC face high recurrence and metastasis rates due to residual micrometastatic lesions, incomplete tumor removal, and postoperative immunosuppression, resulting in a 5-year survival rate of only 41% [2].

Adjuvant therapies, including chemotherapy, radiotherapy, and targeted therapies, aim to reduce recurrence and metastasis. Although chemotherapy and radiotherapy are the standard adjuvant modalities for NSCLC patients, they have limited long-term survival benefits, high recurrence and metastasis rates, and significant toxicity. Studies such as the Lung Adjuvant Cisplatin Evaluation (LACE) meta-analysis show a modest improvement in 5-year survival rates with platinum-based regimens [3]. Newer treatments, including targeted therapies for EGFR (Epidermal growth factor receptor) and ALK (Anaplastic lymphoma kinase) mutations, as well as immunotherapy, have demonstrated survival benefits, but targeted therapies are limited to EGFR and ALK mutant patients, and immunotherapy shows promising applications for patients with high PD-L1 expression [4–7]. Thus, there remains a need for effective, accessible, and well-tolerated treatments for postoperative stage IIIA NSCLC patients.

Multidisciplinary treatment combining traditional Chinese medicine (TCM) and Western medicine has become increasingly integrated into cancer care in China, focusing on strengthening the body’s vital energy (Zheng Qi) to enhance immunity and reduce recurrence and metastasis risks [8]. TCM approaches, such as herbal treatments based on syndrome differentiation, have shown promise in improving survival outcomes and quality of life [9–13]. Despite growing interest, there is a lack of high-quality real-world evidence on the effectiveness of TCM focused on strengthening the body (Fu Zheng Pei Ben) in preventing postoperative recurrence and metastasis in stage IIIA NSCLC, particularly regarding treatment duration, subgroup analysis, and recurrence/metastasis sites.

This study aims to evaluate the impact of TCM therapy focused on strengthening the body on preventing recurrence and metastasis in postoperative stage IIIA NSCLC patients based on real-world data.

## Methods

### Study design and population

This retrospective cohort study utilized real-world data from patients diagnosed and treated at both outpatient and inpatient departments of Longhua Hospital, affiliated with Shanghai University of Traditional Chinese Medicine, between January 2016 and January 2022. Patients included in the study had a histological or cytological diagnosis of stage IIIA NSCLC following surgery and adjuvant chemotherapy or radiotherapy. Clinical data were extracted from the hospital's HIS system to establish a study database. The study was approved by the Ethics Committee of Longhua Hospital (Ethics Approval No. 2022LCSY029) and adhered to the Declaration of Helsinki. As a retrospective study utilizing de-identified data for analysis, informed consent was waived.

Patients were eligible for inclusion if they: (1) Were diagnosed with stage IIIA NSCLC and underwent radical surgery; (2) Had no recurrence or metastasis within 3 months after surgery; (3) Had a TCM syndrome differentiation of Qi deficiency, Yin deficiency, or Qi-Yin deficiency; (4) Were aged between 18 and 75 years (inclusive); (5) Had clear clinical pathological staging; (6) Had an Eastern Cooperative Oncology Group (ECOG) performance status (PS) of ≤ 3; and (7) had normal blood counts as well as normal liver and kidney function; and (8) Had available clinical data.

Patients were excluded if they: (1) received targeted therapy, immunotherapy, or did not receive any adjuvant therapy; (2) Had an expected survival of less than 3 months; (3) had severe comorbidities such as heart failure, liver cirrhosis, or renal insufficiency; or (4) Were pregnant or lactating.

### Exposure

Patients were categorized into exposed and non-exposed groups based on the use of Fu Zheng TCM treatment. The non-exposed group received only adjuvant chemotherapy or radiotherapy. According to Chinese Society of Clinical Oncology (CSCO) Primary Lung Cancer Diagnosis and Treatment Guidelines 2016.V1, chemotherapy followed standard platinum-based doublet regimens for four cycles, each lasting 21 days. Optional adjuvant chemotherapy regimens included: vinorelbine/paclitaxel/docetaxel/pemetrexed (non squamous cell carcinoma)/gemcitabine + cisplatin/carboplatin. Radiotherapy was administered to patients with N2 disease (mediastinal lymph node metastasis).

The exposed group received TCM treatment focused on strengthening the body alongside chemotherapy or radiotherapy. TCM prescriptions were individualized based on syndrome differentiation and primarily focused on Fu Zheng Pei Ben therapy to replenish the body’s vital energy. TCM syndrome differentiation [14, 15] was based on specific symptoms observed in the patients. Qi Deficiency Syndrome was characterized by primary symptoms such as cough with white sputum, fatigue, and a pale tongue, with secondary symptoms including spontaneous sweating, loose stools, and a slippery pulse. Yin Deficiency Syndrome presented with primary symptoms like dry cough with little or bloody sputum, dry mouth/nose, and a red tongue with little coating, with secondary symptoms such as night sweating, insomnia, and a rapid pulse. Qi-Yin Deficiency Syndrome included primary symptoms like cough with scant sputum, fatigue, and dry mouth with little desire to drink, while secondary symptoms could include spontaneous sweating, night sweating, a red or pale tongue, and a weak pulse. It is important to note that the syndrome type may evolve during treatment, which could complicate the assessment of its impact on disease-free survival (DFS). This variability should be considered a limitation when evaluating long-term outcomes in the study.

The exposed group received one of the three prescriptions (Yiqi Jianpi Fang, Yangyin Shengjin Fang, or Yiqi Yangyin Fang) daily, according to their specific syndrome. The Yi Qi Jian Pi Decoction, used for Qi deficiency, contained Radix Astragali (15 g), *Codonopsis* *pilosula* (15 g), *Rhizoma* *Atractylodis* *Macrocephalae* (9 g), Poria (15 g), *Selaginella* *doederleinii* (30 g), *Salvia* *Chinensis* (30 g), *Paris* *polyphylla* (15 g), and other herbs. For Yin deficiency, the Yang Yin Sheng Jin Decoction was used, which included Radix Glehniae (30 g), Radix Adenophorae (30 g), Radix Ophiopogonis (15 g), Radix Asparagi (15 g), *Selaginella* *doederleinii* (30 g), Salvia Chinensis (30 g), *Paris* *polyphylla* (15 g), and additional components. For patients with a combination of Qi and Yin deficiency, the Yi Qi Yang Yin Decoction was prescribed, containing Radix Astragali (30 g), *Rhizoma* *Atractylodis* *Macrocephalae* (9 g), Radix Glehniae (15 g), Radix Ophiopogonis (15 g), *Selaginella* *doederleinii* (30 g), *Salvia* *Chinensis* (30 g), Paris polyphylla (15 g), and other herbs. These prescriptions were supplemented by additional components aimed at heat-clearing, detoxifying, and resolving phlegm. Chinese medicine for strengthening the body is provided by the herbal medicine pharmacy of Longhua Hospital, affiliated with Shanghai University of TCM. Patients could choose to have the pharmacy decoct the medicine for them or decoct it themselves. When patients chose to decoct it themselves, they were informed of the decoction method. Pour the traditional Chinese medicine into the medicine jar, soak it in warm water for 2 h, then start frying the first herbal soup. After boiling, fry for 30–40 min. Fry the second herbal soup for 30–40 min, then remove the residue. Combine the first and second herbal soup together and fry until 500 mL. One dose of Chinese medicine was 500 mL per day, which were taken warm twice daily, in the morning and evening after meals. A treatment cycle was defined as 28 days, with a minimum of three cycles, consistent with standard practices in TCM for cancer treatment, where a continuous treatment period of 2–3 months is typically required to observe therapeutic effects [16].

### Outcomes and data collection

Following baseline investigations, all participants entered the follow-up phase. Regular follow-up evaluations included chest CT scans, cranial MRI, abdominal CT, and bone scans. Disease recurrence, metastasis, and survival status were monitored every 3 months until disease recurrence/metastasis occurred or the study concluded.

The primary outcome [17] was DFS, defined as the time from enrollment to disease recurrence/metastasis or death from any cause. DFS was calculated based on RECIST criteria, measured in months. For participants who remained recurrence-free at study completion or were lost to follow-up, DFS was censored at the date of their last tumor evaluation. For patients without recurrence or metastasis, the last imaging evaluation date was used as the censoring date.

The secondary outcomes [17] included the 1-year, 2-year, and 3-year disease-free survival rates (DFSR), which represent the proportion of patients who remained free of recurrence or metastasis at 1, 2, and 3 years postoperatively, expressed as percentages. The time from enrollment to recurrence or metastasis was recorded, and DFS rates were calculated for each time point.

Clinical characteristics were retrieved from the hospital’s medical record management system, including demographic data (sex, age), tumor characteristics [T-stage, tumor length, N-stage, pleural invasion, vascular cancer embolism, nerve invasion, and spread through air spaces (STAS)], pathological type, and treatment-related factors (completion of four chemotherapy cycles, N2 radiotherapy).

### Statistical analysis

All clinical data were entered into a study database. Statistical analyses were performed using SPSS 26.0 (IBM Corp., Armonk, NY, USA) and R 4.3.2 (R Foundation for Statistical Computing, Vienna, Austria) software. To reduce potential bias, propensity score matching (PSM) was applied using a 1:1 ratio between the exposed and non-exposed groups. Comparisons of demographic and baseline characteristics were performed using variance analysis or chi-square tests to ensure intergroup balance. Kaplan–Meier survival analysis with log-rank tests was conducted to compare survival outcomes. The 1-, 2-, and 3-year DFS rates were analyzed using the life table method, with Z-tests applied for significance testing.

Univariate and multivariate Cox regression analyses were performed to analyze the factors affecting postoperative recurrence and metastasis in stage IIIA NSCLC. The hazard ratio (HR) for the variables was calculated. Potential risk/protective factors for postoperative recurrence and metastasis of NSCLC, including sex, age, T stage, N stage, pathological type, pleural invasion, intravascular tumor thrombus, nerve invasion, STAS, tumor length greater than 4 cm, completion of 4 courses of chemotherapy, and N2 radiotherapy, were included as covariates in the Cox regression model. Variables with a *p*-value < 0.05 in univariate analysis were included in the multivariate regression analysis to identify independent risk factors for postoperative recurrence and metastasis. A two-sided *p*-value < 0.05 was considered statistically significant.

## Results

### Basic characteristics

**Fig. 1:**
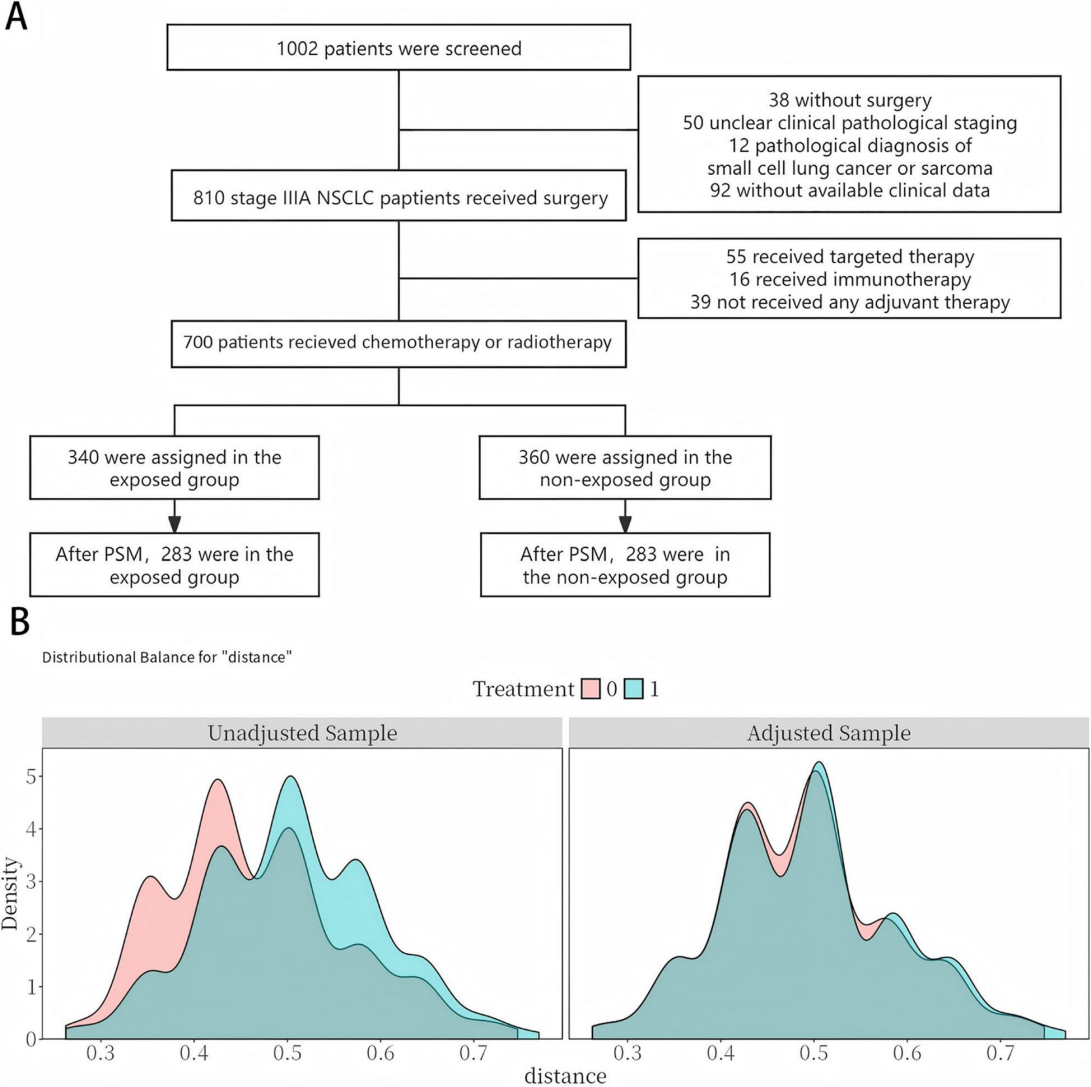
**A** Flowchart of this study. **B** Probability density distributions before and after PSM. 0 means the non-exposed Group; 1 means the exposed group

A total of 1002 patients were screened for eligibility. Among them, 38 patients did not undergo surgery, 50 had unclear clinical pathological staging, 12 were diagnosed with small cell lung cancer or sarcoma, and 92 lacked available clinical data. As a result, 810 patients with stage IIIA NSCLC who had undergone surgery were identified. Of these, 55 received targeted therapy, 16 received immunotherapy, and 39 did not receive adjuvant therapy, leading to their exclusion. Therefore, 700 patients were enrolled, with 340 in the exposed group, and 360 in the non-exposed group. After PSM, 283 patients remained in each group (Fig. 1A, B).

Baseline characteristics, such as sex, age, T-stage, tumor length > 4 cm, N-stage, pleural invasion, vascular cancer embolism, nerve invasion, STAS, pathological type, completion of four chemotherapy cycles, and radiotherapy for N2 patients, showed no significant differences between the exposed and non-exposed groups (all *p* > 0.05), indicating baseline comparability between the two groups (Table 1).

**Table 1:** Baseline characteristics of patients before and after PSM

	Before PSM				After PSM			
	Total (n = 700)	Non-exposed group (n = 360)	Exposed group (n = 340)	*p*	Total (n = 566)	Non-exposed group (n = 283)	Exposed group (n = 283)	*p*
Sex, n (%)				0.104				0.865
Male	283 (40.43)	135 (37.50)	148 (43.53)		238 (42.05)	120 (42.40)	118 (41.70)	
Female	417 (59.57)	225 (62.50)	192 (56.47)		328 (57.95)	163 (57.60)	165 (58.30)	
Age, n (%)				0.097				0.653
< 65 years	462 (66)	248 (68.89)	214 (62.94)		383 (67.67)	189 (66.78)	194 (68.55)	
≥ 65 years	238 (34)	112 (31.11)	126 (37.06)		183 (32.33)	94 (33.22)	89 (31.45)	
T-stage, n (%)				0.900				0.341
T1–2	547 (78.14)	282 (78.33)	265 (77.94)		455 (80.39)	223 (78.80)	232 (81.98)	
T3–4	153 (21.86)	78 (21.67)	75 (22.06)		111 (19.61)	60 (21.20)	51 (18.02)	
Tumor length > 4 cm, n (%)				0.145				0.370
No	520 (74.29)	259 (71.94)	261 (76.76)		435 (76.86)	213 (75.27)	222 (78.45)	
Yes	180 (25.71)	101 (28.06)	79 (23.24)		131 (23.14)	70 (24.73)	61 (21.55)	
N-stage, n (%)				0.061				0.510
N0–1	90 (12.86)	38 (10.56)	52 (15.29)		65 (11.48)	35 (12.37)	30 (10.60)	
N2	610 (87.14)	322 (89.44)	288 (84.71)		501 (88.52)	248 (87.63)	253 (89.40)	
Pleural invasion, n (%)				0.087				0.861
No	399 (57)	194 (53.89)	205 (60.29)		362 (63.96)	180 (63.60)	182 (64.31)	
Yes	301 (43)	166 (46.11)	135 (39.71)		204 (36.04)	103 (36.40)	101 (35.69)	
Vascular cancer embolismn, n (%)				0.043				0.494
No	534 (76.29)	286 (79.44)	248 (72.94)		427 (75.44)	217 (76.68)	210 (74.20)	
Yes	166 (23.71)	74 (20.56)	92 (27.06)		139 (24.56)	66 (23.32)	73 (25.80)	
Nerve invasion, n (%)				0.215				0.362
No	679 (97)	352 (97.78)	327 (96.18)		546 (96.47)	275 (97.17)	271 (95.76)	
Yes	21 (3)	8 (2.22)	13 (3.82)		20 (3.53)	8 (2.83)	12 (4.24)	
STAS, n (%)				0.098				0.459
No	612 (87.43)	322 (89.44)	290 (85.29)		490 (86.57)	248 (87.63)	242 (85.51)	
Yes	88 (12.57)	38 (10.56)	50 (14.71)		76 (13.43)	35 (12.37)	41 (14.49)	
Pathological type, n (%)				0.230				0.255
Squamous cell carcinoma	114 (16.29)	63 (17.50)	51 (15.00)		81 (14.31)	47 (16.61)	34 (12.01)	
Adenocarcinoma	550 (78.57)	283 (78.61)	267 (78.53)		459 (81.1)	222 (78.45)	237 (83.75)	
Others	36 (5.14)	14 (3.89)	22 (6.47)		26 (4.59)	14 (4.95)	12 (4.24)	
Completion of 4 chemotherapy cycles, n (%)				0.683				0.051
No	86 (12.29)	46 (12.78)	40 (11.76)		67 (11.84)	41 (14.49)	26 (9.19)	
Yes	614 (87.71)	314 (87.22)	300 (88.24)		499 (88.16)	242 (85.51)	257 (90.81)	
Radiotherapy for N2 patients, n (%)				0.267				0.818
No	418 (68.52)	227 (70.50)	191 (66.32)		341 (68.06)	170 (68.55)	171 (67.59)	
Yes	192 (31.48)	95 (29.50)	97 (33.68)		160(31.94)	78 (31.45)	82 (32.41)	
Gene mutation for adenocarcinoma patients, n (%)				0.115				0.106
EGFR/ALK/ROS1	216 (39.27)	103 (36.40)	113 (42.32)		180 (39.22)	79 (35.59)	101 (42.62)	
Other mutations	50 (9.10)	29 (10.25)	21 (7.87)		42 (9.15)	21 (9.46)	21 (8.86)	
No mutation	106 (19.27)	64 (22.61)	42 (15.73)		89 (19.39)	53 (23.87)	36 (15.19)	
Not conducted	178 (32.36)	87 (30.74)	91 (34.08)		148 (32.24)	69 (31.08)	79 (33.33)	

*STAS* spread through air spaces, *ROS1*, ROS proto-oncogene 1, receptor tyrosine kinase

### Survival analysis

**Fig. 2:**
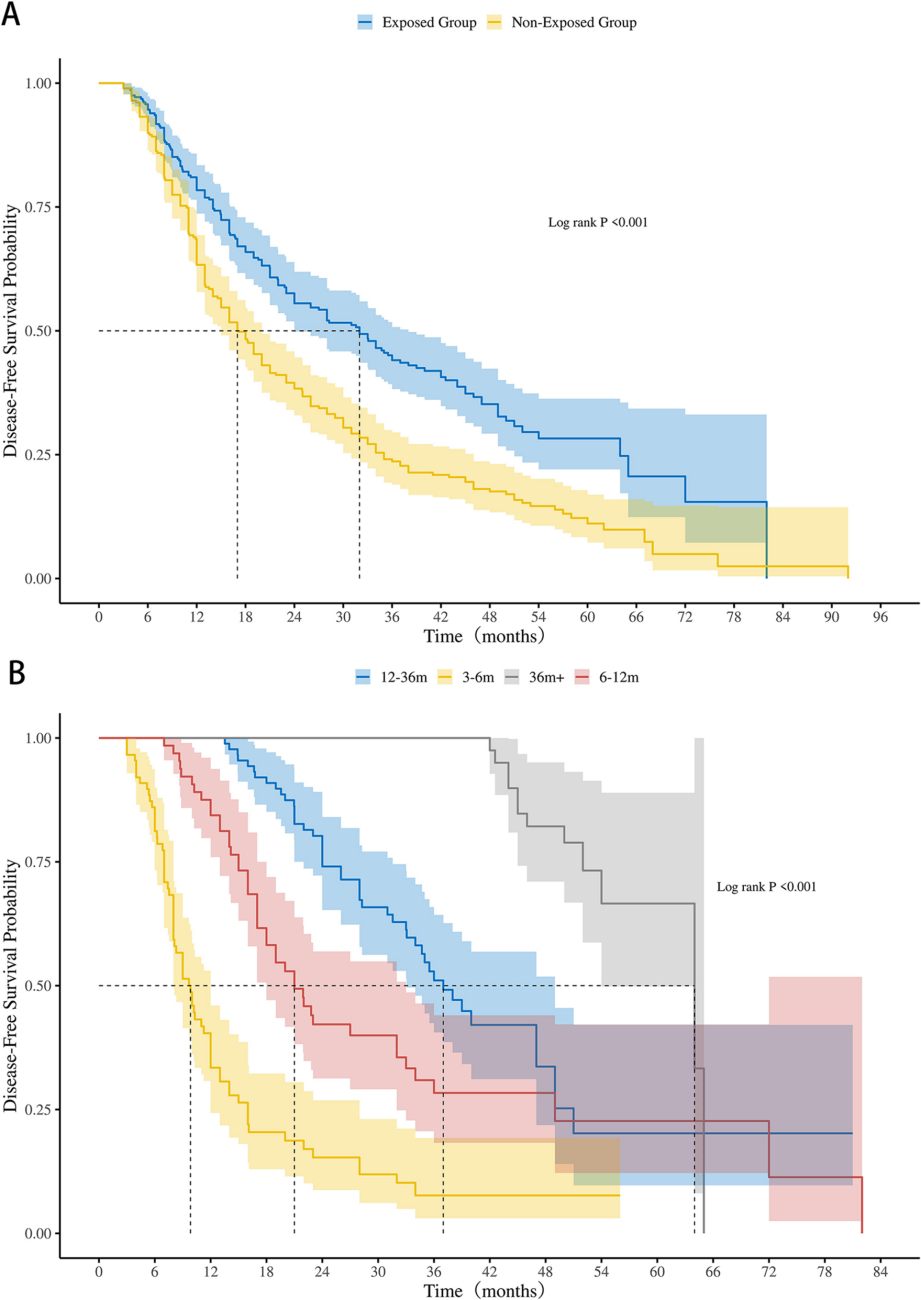
**A** Kaplan–Meier analysis of DFS in the exposed and non-exposed groups. **B** Kaplan–Meier analysis of DFS in groups with different durations of TCM treatment

As of December 31, 2024, with an average follow-up time of 40.0 months, 397 out of 566 patients experienced disease recurrence or metastasis. The recurrence rate was significantly lower in the exposed group (59.36%, 168/283 cases) compared to the non-exposed group (80.92%, 229/283 cases). The median DFS in the exposed group was 32.0 months (95% CI 24.0–38.0), significantly longer than the 17.0 months (95% CI 15.0–20.0) observed in the non-exposed group (*p* < 0.001) (Fig. 2A). Furthermore, the exposed group demonstrated superior 1-year, 2-year, and 3-year DFS rates compared to the non-exposed group (78% vs. 63%, 56% vs. 38%, and 44% vs. 24%, respectively; *p* < 0.001). The univariate and multivariate analysis showed that TCM therapy (exposure) was associated with postoperative recurrence and metastasis in patients with stage IIIA NSCLC (HR = 0.58, 95% CI 0.48–0.70, *p* < 0.001) (Table 2).

**Table 2:** MultivariateCox proportional hazards model

	Unadjusted					Fully Adjusted				
	β	S.E	Z	*p*	HR (95%CI)	β	S.E	Z	*p*	HR (95%CI)
**TCM**										
No					1.00 (Reference)					1.00 (Reference)
Yes	− 0.52	0.10	− 5.11	< 0.001	0.59 (0.49–0.73)	− 0.54	0.10	− 5.26	**< 0.001**	0.58 (0.48–0.71)

The multivariate Cox proportional hazards model adjusted for demographic factors (sex, age), tumor characteristics (T-stage, tumor length, N-stage, pleural invasion, vascular cancer embolism, nerve invasion, and STAS), pathological type, and treatment-related factors (completion of four chemotherapy cycles and radiotherapy for N2 patients)

*TCM* traditional Chinese medicine, *HR* Hazard Ratio, *CI* Confidence Interval

Among patients with recurrence, the exposed group exhibited fewer stage IV cases compared to the non-exposed group (140 vs. 203 cases). The exposed group also showed lower incidences of lung metastasis, mediastinal metastasis, pleural metastasis, lymph node metastasis, liver metastasis, and bone metastasis (*p* < 0.05). Additionally, the exposed group had fewer occurrences of recurrence in critical organs. These findings highlight a trend toward reduced metastasis severity and spread in the exposed group.

In the exposed group, patients were categorized based on the duration of TCM treatment into 3–6 months, 6–12 months, 12–36 months, and over 36 months groups. A longer duration of TCM treatment was associated with progressively improved DFS outcomes. The median DFS for patients receiving treatment for 6–12 months, 12–36 months, and over 36 months were 21.0 months (95% CI 18.0–33.0), 37.0 months (95% CI 33.1–49.0), and 64.0 months (95% CI 54.0–NA), respectively, all significantly better than the 9.8 months (95% CI 8.0–12.0) observed in the 3–6 months group (*p* < 0.001) (Fig. 2B).

### Subgroup analyses

**Fig. 3:**
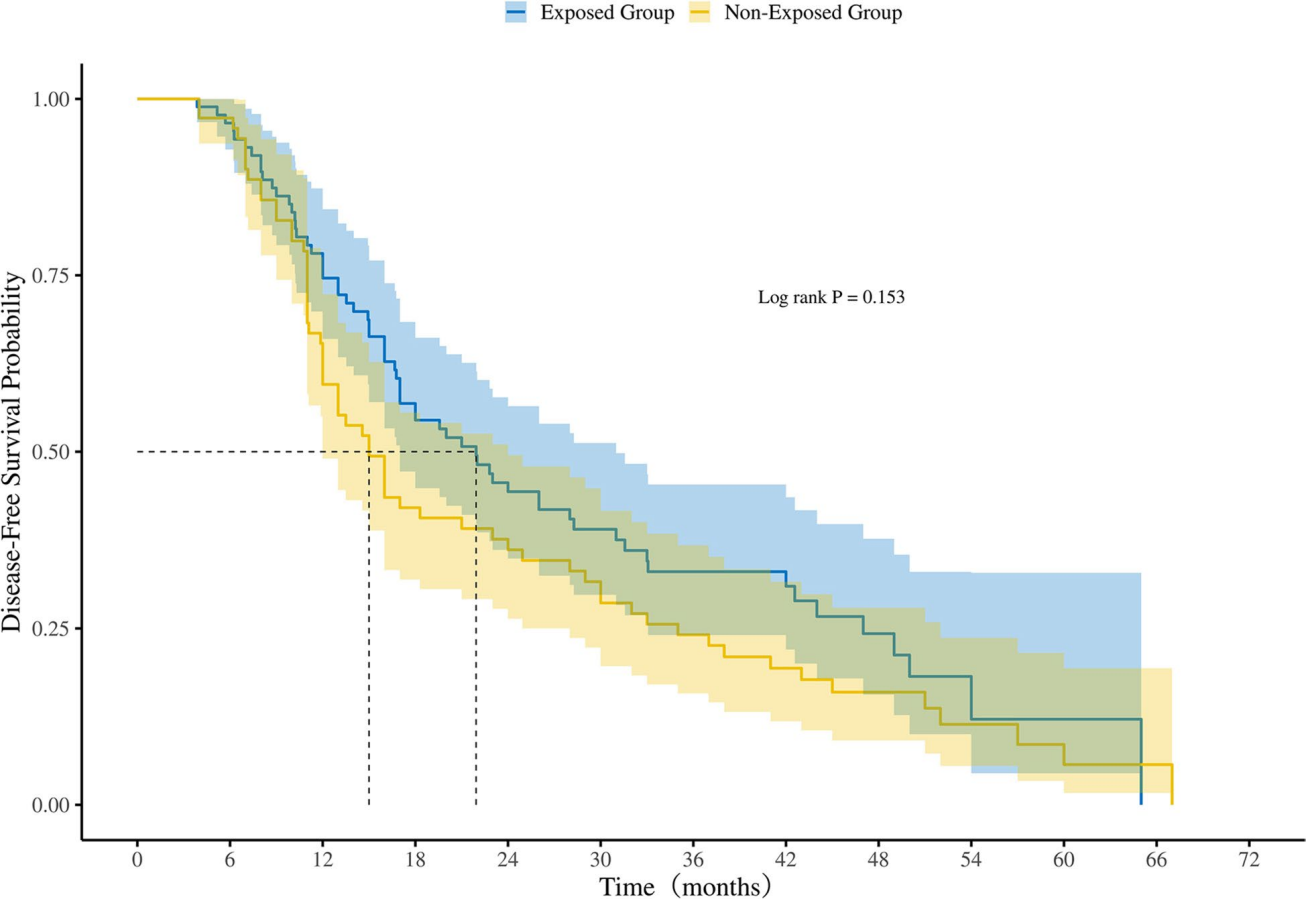
Kaplan–Meier analysis of DFS in the EGFR mutation subgroup

Among the 566 patients, 311 adenocarcinoma patients (158 in the exposed group, 153 in the non-exposed group) underwent genetic testing. 163 patients (89 in the exposed group, 74 in the non-exposed group) had an EGFR mutation. In the EGFR mutation subgroup, the median DFS was 21.9 months (95% CI 17.0–31.0) in the exposed group compared to 15.0 months (95% CI 12.0–24.0) in the non-exposed group, with no statistically significant difference (*p* = 0.153) (Fig. 3).

**Fig. 4:**
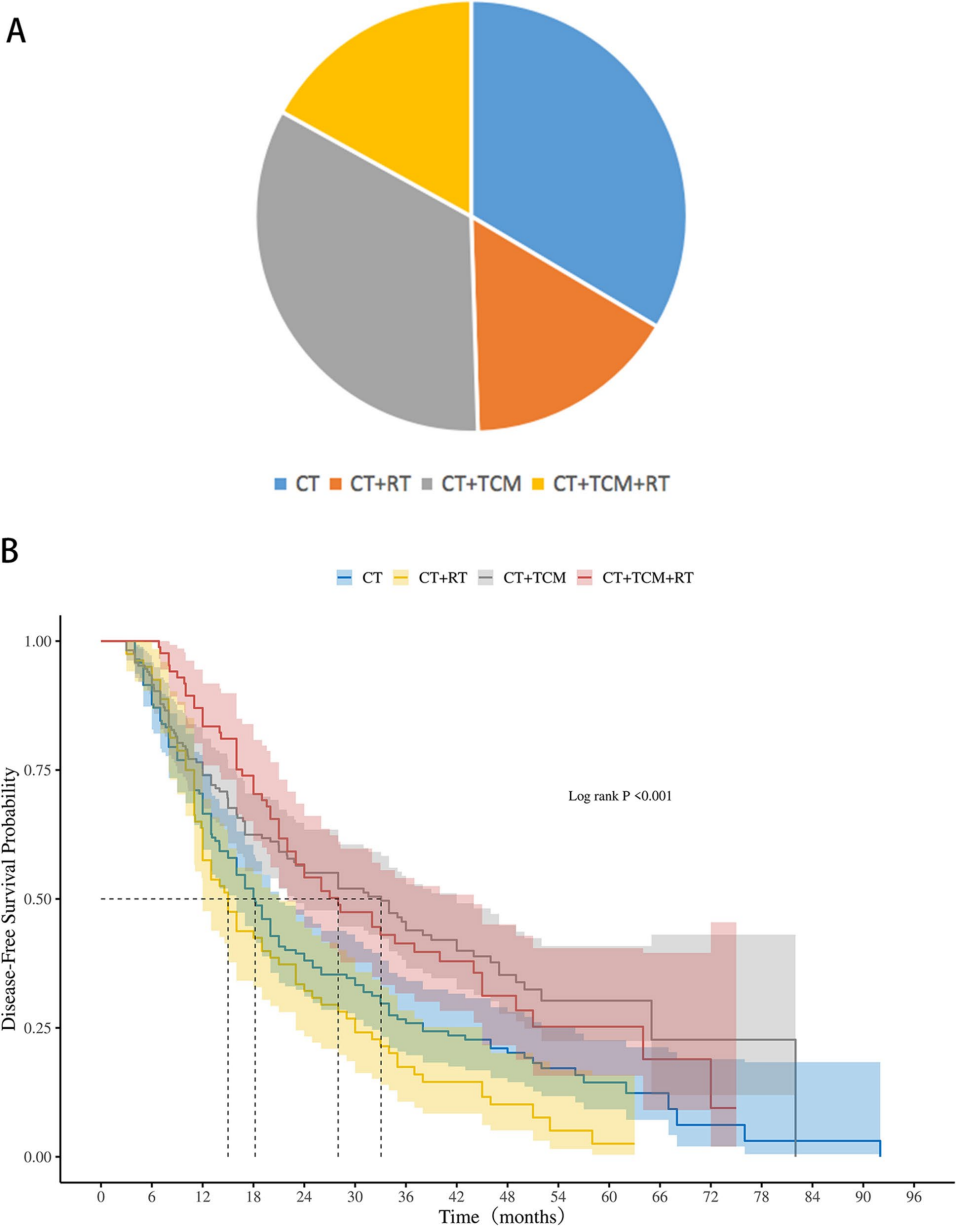
**A** Treatment strategies in the N2 subgroup. **B** Kaplan–Meier Analysis of DFS in the N2 Subgroup. *CT* chemotherapy, *RT* radiotherapy, *TCM* Traditional Chinese Medicine

For patients with N2 involvement, treatment strategies included chemotherapy, chemotherapy combined with radiotherapy, chemotherapy combined with TCM medicine, and a combination of all three modalities (Fig. 4A). Radiotherapy alone did not significantly extend DFS, with median DFS times of 18.2 months (95% CI 16.0–21.0) in the chemotherapy group and 15.0 months (95% CI 12.0–21.0) in the chemotherapy + radiotherapy group (*p* = 0.068). However, the addition of TCM medicine significantly improved DFS outcomes. Patients receiving chemotherapy + TCM medicine had a median DFS of 33.1 months (95% CI 22.8–42.0), while those treated with chemotherapy + radiotherapy + TCM medicine achieved a median DFS of 28.0 months (95% CI 22.0–44.0). Both comparisons showed significant differences compared to the groups without TCM medicine (*p* < 0.001) (Fig. 4B).

**Fig. 5:**
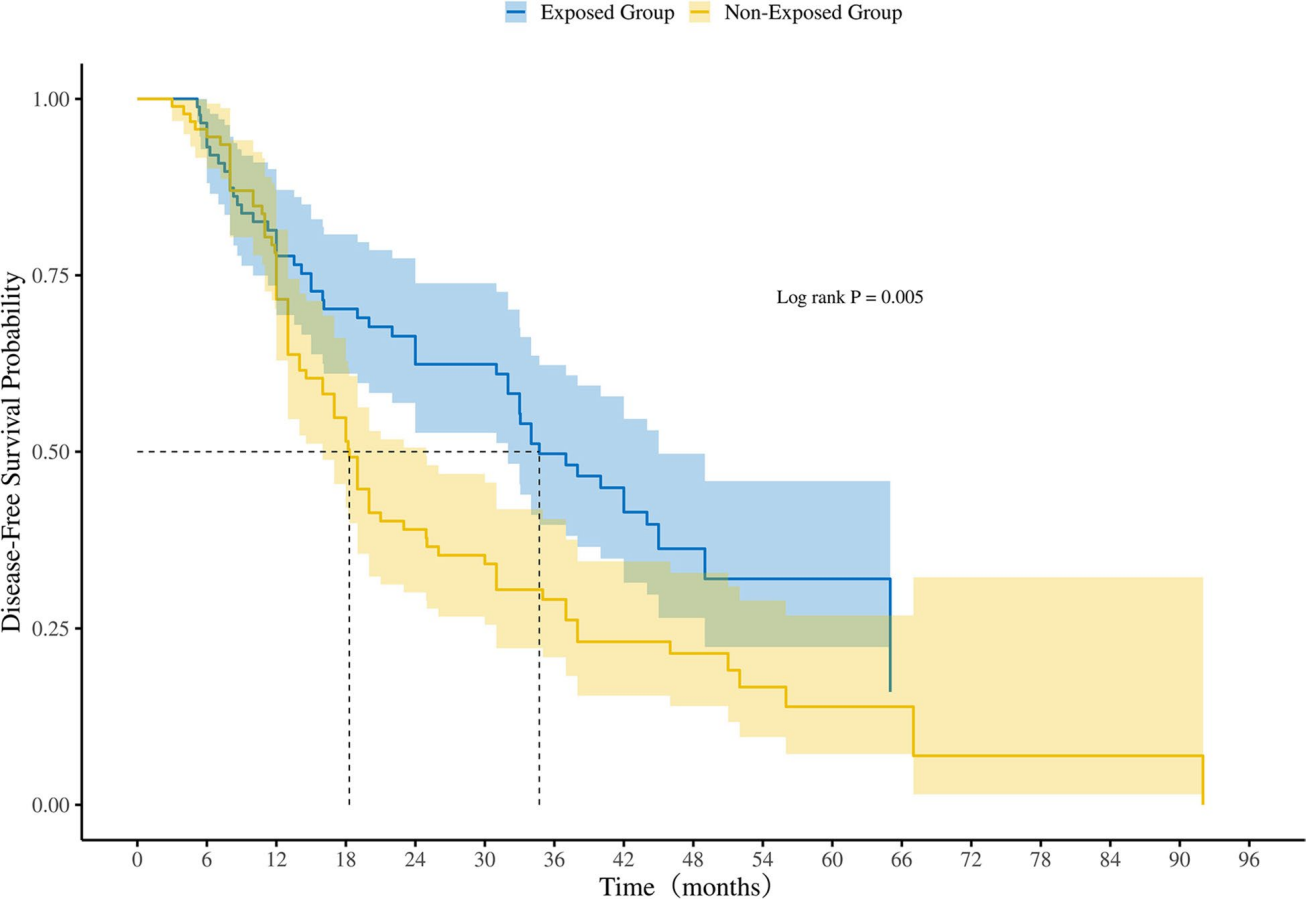
Kaplan–Meier analysis of DFS in patients aged over 65 years

In the subgroup of patients aged over 65 years, the exposed group demonstrated a significantly longer median DFS of 34.7 months (95% CI 32.0–45.0) compared to 18.3 months (95% CI 16.0–24.9) in the non-exposed group (*p* = 0.005) (Fig. 5).

**Fig. 6:**
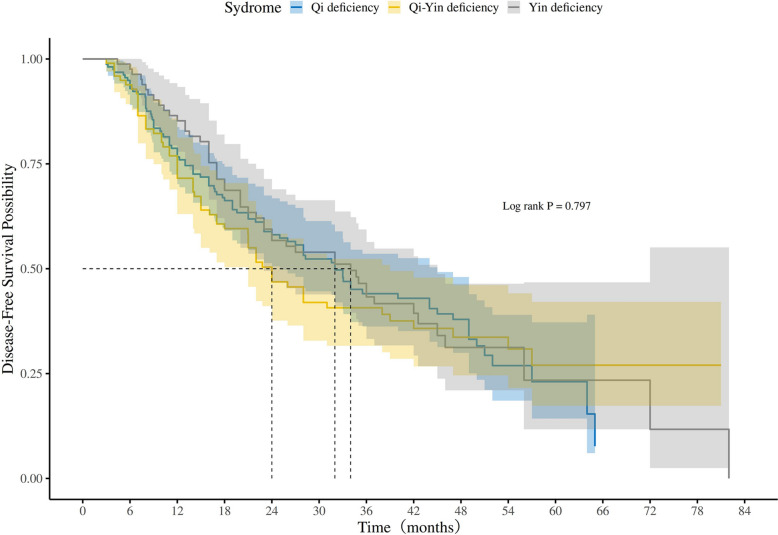
Kaplan–Meier analysis of DFS in patients with different syndrome

In the exposed group, the median DFS of patients with Qi deficiency syndrome, Yin deficiency syndrome, and Qi-Yin syndrome were 32.0 months (95% CI 25.0–45.0), 34.0 months (95% CI 23.0–45.0), and 24.0 months (95% CI 21.0–39.0), with no significant difference (p = 0.797) (Fig. 6).

## Discussion

This study provides strong evidence supporting the role of TCM therapy focused on strengthening the body as an effective adjuvant treatment for postoperative stage IIIA NSCLC patients. The main results show that TCM therapy focused on strengthening the body significantly extended DFS, particularly in the N2 subgroup and elderly patients. Additionally, it was associated with lower rates of recurrence and metastasis. These findings suggest that TCM therapy focused on strengthening the body, integrated with standard chemoradiotherapy, may play a critical role in preventing recurrence and metastasis in postoperative NSCLC patients.

The results of this study align with a growing body of research demonstrating the benefits of combining TCM with Western treatments, particularly in improving outcomes for NSCLC patients. For instance, Guo et al. showed that herbal treatments significantly improve the quality of life in metastatic NSCLC patients undergoing platinum-based chemotherapy, suggesting that TCM can alleviate symptoms and enhance patient well-being during aggressive treatments [18]. Similarly, Zhao et al. reported that TCM integration with chemotherapy in stage II-IIIA NSCLC patients after radical surgery led to improved survival outcomes, with a notable reduction in recurrence rates and enhanced DFS [19]. Our study supports these findings, showing that TCM therapy focused on strengthening the body not only extended DFS but also improved the 1-year, 2-year, and 3-year DFS rates compared to those receiving chemoradiotherapy alone. Moreover, the duration of TCM therapy emerged as an important factor in improving survival outcomes. Wang et al. similarly found that longer TCM treatment durations were associated with better survival in NSCLC patients, highlighting the potential cumulative benefits of sustained TCM therapy [20]. This pattern was replicated in our study, where longer durations of TCM treatment were correlated with progressively better DFS outcomes.

In addition to enhancing DFS, TCM has also been shown to support chemotherapy by modulating immune function and reducing adverse effects. For example, Li et al. conducted a meta-analysis showing that Chinese herbal medicine combined with chemotherapy significantly increased the 1-year survival rate in patients with advanced NSCLC, supporting the beneficial role of TCM in improving patient prognosis [11]. However, not all studies report consistent survival benefits. Xiao et al. found that while TCM improved quality of life and symptom control during chemotherapy, it did not significantly impact survival outcomes, suggesting that its effectiveness may vary depending on the therapeutic approach or patient population [21]. These contrasting results underscore the importance of TCM treatment selection, duration, and integration strategy. Our findings suggest that when appropriately applied, TCM can contribute to prolonged DFS, highlighting its potential as a valuable adjunct to chemoradiotherapy in NSCLC management.

Furthermore, a study by Wu et al. found that combining TCM with concurrent chemoradiotherapy led to improved local control and reduced metastasis in NSCLC patients [22]. Our study found similar results, with patients receiving chemotherapy plus TCM showing a significantly longer median DFS compared to those receiving chemotherapy alone, suggesting that TCM not only supports chemotherapy but may also enhance the efficacy of radiation therapy. This aligns with the work of Chen et al., who highlighted the potential of TCM to modulate immune responses and reduce inflammation, which may contribute to the suppression of tumor progression [23].

A review by Yang et al. also emphasized TCM's ability to reduce chemotherapy-induced toxicity, improve immune function, and improve survival outcomes in various cancer types [24]. While the studies reviewed above differ in the specific combination of therapies used and the types of TCM treatments administered, the overall consensus supports the potential synergistic effects of TCM when integrated with Western treatments for cancer. Our study contributes to this evidence, particularly in NSCLC, where TCM has shown promise in reducing recurrence and metastasis, improving DFS for patients.

In summary, the results of our study further strengthen the evidence that TCM therapy can be a valuable adjunct to Western treatments in improving outcomes for NSCLC patients, particularly in terms of DFS. However, more research is needed to identify optimal treatment regimens, understand the underlying molecular mechanisms, and confirm the clinical benefits of TCM in larger, multicenter randomized controlled trials.

Regarding the subgroup analysis, our study highlighted that patients with EGFR mutations did not show significant improvement in DFS with Fu-Zheng therapy. EGFR-TKI targeted therapies are highly effective in extending DFS in EGFR-mutant NSCLC patients [4, 5, 25]. Future research should explore the potential role of TCM as an adjunct to these targeted therapies.

In the N2 subgroup, while radiotherapy did not significantly impact DFS in our study, previous studies such as those by Lally et al. and Robinson et al. found that postoperative radiotherapy improved overall survival (OS) for patients with mediastinal lymph node involvement [26–28]. However, studies like the PORT-C trial and the Lung ART trial suggested that radiotherapy may improve local control rates but does not impact distant metastasis [29, 30]. Our study found that Fu-Zheng therapy extended DFS in patients with stage IIIA-N2 disease regardless of whether they received adjuvant radiotherapy, further supporting the role of TCM in preventing recurrence.

Additionally, for elderly patients, our study showed that prolonged TCM treatment was associated with longer DFS, consistent with the findings of You et al. [31], who demonstrated that the integration of Chinese herbal medicine with conventional treatment significantly improved overall survival in patients with NSCLC. Fu-Zheng therapy helped reduce metastasis rates to lung, mediastinum, pleura, lymph node, liver and bone, contributing to the reduction in stage IV progression.

This study has several limitations. First, it is a retrospective cohort study, which inherently carries the risk of selection bias. Prospective, multi-center studies with larger sample sizes are necessary to validate these findings and provide more reliable data. Furthermore, the study focused solely on DFS and did not assess other outcomes such as overall survival (OS) or the potential long-term impacts of Fu-Zheng therapy. A significant limitation is the inclusion of only patients who received adjuvant chemotherapy (radiotherapy) before 2022, as patients with EGFR mutations after this time began to receive adjuvant targeted therapy. The study's design intentionally excludes those receiving such targeted therapies after 2022 to ensure a consistent patient population for comparison. Additionally, while this study provides valuable evidence for the integration of TCM with Western medicine, the exact mechanisms by which Fu-Zheng therapy prevents recurrence and metastasis remain unclear and warrant further investigation.

## Conclusion

In conclusion, this retrospective cohort study provides evidence for the role of Fu-Zheng therapy as an effective adjuvant treatment for postoperative stage IIIA NSCLC patients. Fu-Zheng therapy significantly extended DFS, particularly in the N2 subgroup and elderly patients, and was associated with lower rates of recurrence and metastasis. While the findings support the clinical integration of TCM with Western medicine in the management of stage IIIA NSCLC, further prospective studies are required to validate these results. Future research should also investigate the potential role of TCM in combination with targeted therapies and immunotherapies and extend its observations to overall survival and biomarker-based monitoring of disease prognosis.

## Data Availability

All data generated or analysed during this study are included in this published article.

## Abbreviations

TCM: Traditional Chinese Medicine
NSCLC: Non-small cell lung cancer
DFS: Disease-free survival
DFSR: DFS rates
EGFR: Epidermal growth factor receptor
ALK: Anaplastic lymphoma kinase
LACE: Lung Adjuvant Cisplatin Evaluation
ECOG: Eastern Cooperative Oncology Group
PS: Performance status
STAS: Spread through air spaces
PSM: Propensity score matching
HR: Hazard ratio
OS: Overall survival
